# Detection of minimal residual disease and prediction of recurrence in breast cancer using a plasma-only circulating tumor DNA assay

**DOI:** 10.1016/j.esmoop.2025.104296

**Published:** 2025-03-22

**Authors:** W. Janni, B. Rack, T.W.P. Friedl, A.D. Hartkopf, L. Wiesmüller, K. Pfister, F. Mergel, A. Fink, T. Braun, F. Mehmeti, N. Uhl, A. De Gregorio, J. Huober, T. Fehm, V. Müller, T.A. Rich, D.J. Dustin, S. Zhang, S.T. Huesmann

**Affiliations:** 1 Department of Obstetrics and Gynecology, University Hospital Ulm, Ulm, Germany; 2 Department of Obstetrics and Gynecology, University Hospital Tübingen, Tübingen, Germany; 3 Department of Obstetrics and Gynecology, Klinikum am Gesundbrunnen, Heilbronn, Germany; 4 Breast Cancer Center St. Gallen, St. Gallen, Switzerland; 5 Department of Obstetrics and Gynecology, University Hospital Düsseldorf, Düsseldorf, Germany; 6 Department of Obstetrics and Gynecology, University Hospital Hamburg-Eppendorf, Hamburg, Germany; 7 Guardant Health Inc., Redwood City, USA

**Keywords:** early breast cancer, minimal residual disease, MRD, circulating tumor DNA, ctDNA, breast cancer recurrence

## Abstract

**Background:**

Detection of minimal residual disease (MRD) in early breast cancer (EBC) after curative-intent treatment may identify patients at risk for recurrence. Most circulating tumor DNA (ctDNA)-based MRD assays require knowledge of genomic alterations from tumor tissue. However, tissue availability may be limited in some patients. Here, we evaluated sensitivity and specificity for recurrence detection, using a plasma-only ctDNA MRD assay.

**Materials and methods:**

For this pilot study, 47 plasma samples from 38 EBC patients were collected at 12 or 36 months post-diagnosis or at clinical recurrence. ctDNA presence was determined by a custom bioinformatics classifier that identifies tumor-derived somatic variants and methylation profiles specific to individual cancer types using a 5-Mb next-generation sequencing panel.

**Results:**

ctDNA was detected at or before distant recurrence in 11/14 (79%) patients [sensitivity was 85% (11/13) among samples collected within 2 years from recurrence]. Lead time was evaluable in 4/6 (67%) samples collected before distant recurrence with detectable ctDNA and ranged from 3.4 to 18.5 months. ctDNA was not detected in samples from patients without recurrence (*n* = 13).

**Conclusions:**

This study demonstrates the feasibility of MRD detection in EBC using a plasma-only multiomic ctDNA-based approach. Larger studies are ongoing to further validate the clinical performance of the assay and demonstrate its applications.

## Highlights

• A plasma-only multiomic ctDNA assay detected ctDNA in 85% (11/13) of patients within 2 years before recurrence.
• ctDNA was detected in 4/6 patients (67%) before distant recurrence with a 3.4 to 18.5-month lead time.
• No ctDNA was detected in the recurrence-free control group (*n* = 13).

## Introduction

More than 90% of breast cancer (BC) patients are diagnosed early without macroscopic evidence of metastasis; however, micrometastatic disease may lead to eventual overt metastatic recurrence if not eradicated by primary curative-intent therapy.^1^ While most early-stage breast cancers (EBCs) are curable, metastatic BC (MBC) remains a major cause of cancer-related mortality, with over 600 000 deaths globally.^1^^,^^2^ Risk for metastatic recurrence persists for decades.^3^

Treatment of EBC combines surgery, radiotherapy, and systemic treatments, which substantially reduce the risk of distant recurrence, presumably by eradicating minimal residual disease (MRD) not cleared by local treatment.^4^ Currently, selection of treatment regimen depends on tumor size, lymph node involvement and tumor biology, as well as patient-specific factors.^5^ Systemic therapies include chemotherapy, endocrine therapy [for estrogen receptor-positive (ER+) and/or progesterone receptor-positive (PgR+) tumors], and human epidermal growth factor receptor 2 (HER2)-targeted therapy (for HER2-positive tumors); patients with specific high-risk EBC may additionally receive newer approved agents such as checkpoint inhibitors, poly (ADP-ribose) polymerase inhibitors, cyclin-dependent kinase 4/6 inhibitors, or antibody–drug conjugates.^6^

Following EBC treatment, screening for metastasis in asymptomatic patients is not recommended, as prior studies demonstrated that intensive surveillance programs did not improve overall survival. These studies, conducted more than a decade ago, relied on imaging and biomarkers that are inferior to current technologies.^7^^,^^8^ There is interest in revisiting the utility of surveillance and early metastases detection in improving outcomes for MBC patients.

Circulating tumor DNA (ctDNA) can be detected through genomic and epigenomic analysis of cell-free DNA (cfDNA) isolated from plasma. It obtains genomic profiling non-invasively and is emerging as a strong predictive and prognostic biomarker in both MBC and EBC.^9^, ^10^, ^11^, ^12^, ^13^, ^14^, ^15^, ^16^, ^17^ Following treatment, ctDNA allows assessment of MRD, offering a sensitive and specific approach to identify patients at risk of recurrence. It may also provide an insight into therapy response.^9^ Currently, most ctDNA assays require genomic sequencing of tumor tissue. Tumor-agnostic assays, however, offer advantages regarding feasibility, as they do not require tumor tissue and provide faster results.

Here, we evaluate a plasma-only assay which detects genomic and epigenomic signatures in EBC patients to assess its performance in predicting BC recurrence.

## Materials and methods

### Study population

This study retrospectively analyzed plasma samples from female EBC patients in the BRandO BiO registry, a regional multicenter observational registry with longitudinal biobanking for newly diagnosed EBC patients at University Hospital Ulm and 19 affiliated hospitals in Germany.^18^ Blood samples were collected at diagnosis, 12, 36, and 60 months, and at first relapse, with follow-up planned over 10 years. Eligible patients are ≥18 years of age, have a histologically confirmed invasive BC (except sarcoma) treated at a BRandO BiO center, and provide written informed consent for storing clinical data and biobanking of biologic material in accordance with the local ethics committee-approved protocol and the Declaration of Helsinki. Patients with other invasive malignancies within the past 3 years are excluded. Clinical data, recorded by certified oncology centers, include age, sex, date of diagnosis, tumor size, extent and characteristics (e.g. HER2−, ER/PgR−, grading status), oncological treatment, recurrence, and survival details. Whole blood (40 ml) is collected in PAXgene® (BD Biosciences, Franklin Lakes, NJ) tubes. Plasma is isolated and cryopreserved at University Hospital Ulm. The recruitment began in January 2016, targeting 4000 patients.

For this pilot study, 47 plasma samples from 38 EBC patients were analyzed to assess the Guardant Reveal assay’s performance in detecting MRD and predicting future recurrence. Samples were selected to enrich for recurrence events to estimate assay sensitivity. For specificity assessment, this study included recurrence-free control patients at 3-year follow-up. The study involved samples collected at 12 or 36 months post-diagnosis or at clinical recurrence, blinded to recurrence status. Clinical data were locked in January 2023.

### ctDNA detection

The Guardant Reveal multi-cancer assay (v1.3; Guardant Health, Redwood City, CA) is a multiomic next-generation sequencing (NGS)-based test for detecting ctDNA in solid tumors without requiring tissue-informed mutational data. Using automated and proprietary methods, cfDNA is extracted from plasma, quantified, partitioned based on the fragment methylation percentage, and prepared for target capture by high-efficiency NGS-library preparation. Molecular barcoding enables tracking individual molecules for error correction and tracks methylation partitioning during sequencing. This determines methylation status non-destructively, allowing sensitive genomic and epigenomic analysis of the same cfDNA fragments. Library-prepared samples are enriched for a 5.3-Mb panel covering ∼500 genes commonly mutated in solid tumors and ∼4 Mb of epigenomic regions with differential methylation patterns between tumor and normal blood cells. A custom bioinformatics classifier identifies tumor-specific variants and methylation profiles, excluding interferences such as clonal hematopoiesis. A sample is considered positive using indication-specific probabilistic models with a pre-defined calling threshold. Positive calls from either the somatic or epigenomic panel are integrated to a final ctDNA detected status.

Guardant Reveal also estimates the ctDNA fraction in blood. The genomic tumor fraction is the maximum variant allele fraction (VAF), calculated as tumor variant reads divided by total reads (%).

For methylation, the tumor fraction is estimated using a probabilistic model of tumor- and normally derived molecules in differential methylation regions. Samples with undetectable ctDNA are VAF 0%.

### Statistical analysis

Data were summarized using descriptive statistics including medians with range and proportions. Sensitivity was defined as the proportion of patients who had ctDNA detected at or before clinical recurrence among recurring patients, evaluated separately for local and distant recurrence. Specificity was defined as the proportion of patients who had undetectable ctDNA in patients without recurrence. Categorical data were compared using Fisher’s exact test, with *P* ≤ 0.05 considered statistically significant.

## Results

### ctDNA detection

**Figure 1:**
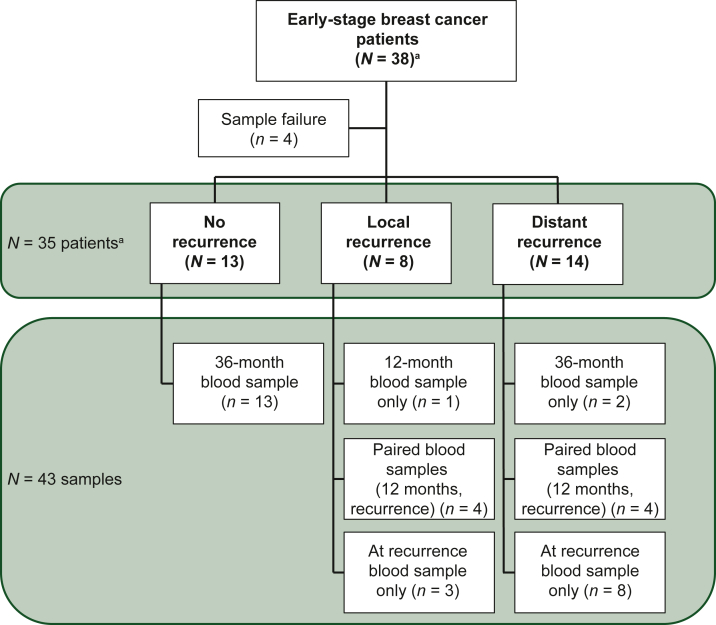
**Breast cancer clinical feasibility pilot composition**. Forty-seven plasma samples from 38 patients with early-stage breast cancer were collected through the BRandO BiO registry at 12 or 36 months post-diagnosis and/or at the time of clinical recurrence. ^a^Selected sequential patients to enrich for recurrences for the purpose of feasibility.

Forty-seven plasma samples (6 ml each; median cfDNA yield 15.1 ng) from 38 patients were tested. The majority (43/47; 91%) of samples met quality requirements and were further analyzed. Of the 35 patients with at least one sample successfully tested, the majority had hormone receptor-positive (HR+)/HER2− BC (71%) and pathological stage I or II disease (63%; Supplementary Table S1, https://doi.org/10.1016/j.esmoop.2025.104296). Fourteen developed distant recurrence, 8 developed local recurrence, and 13 had no evidence of recurrence at 3 years post-diagnosis. The median time from diagnosis to detection of recurrence was 19.2 months (range 3.8-69.9 months), and the median duration of follow-up in patients without recurrence was 37.2 months (range 36.1-66.8 months). Samples were available at the time of recurrence in 19 patients and before recurrence (either at 12 or 36 months post-diagnosis) in 11 patients. All 13 samples from non-recurred patients were collected at 36 months post-diagnosis (Figure 1).

**Figure 2:**
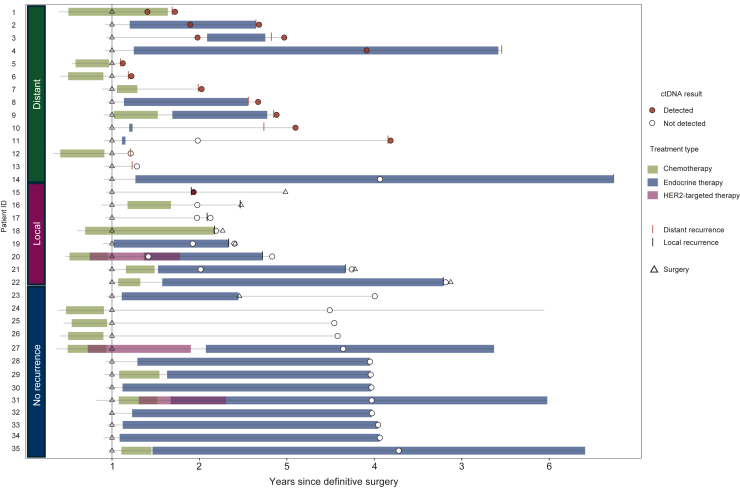
**Swimmer plot outlining timing of treatment, relapse, and ctDNA collection.** ctDNA, circulating tumor DNA; HER2, human epidermal growth factor receptor 2.

In patients with distant recurrence, sensitivity was 79% (11/14; Table 1, Figure 2). Among the subset of samples collected before detection of distant recurrence, 4/6 (67%) had detectable ctDNA with lead times of 3.4, 9.0, 10.1, and 18.5 months. In the two patients in whom ctDNA was not detected before recurrence, the ctDNA-negative samples were collected 26 and 32 months before recurrence. Therefore, the sensitivity for detecting distant recurrence in samples collected within 2 years from recurrence was 85% (11/13). The sensitivity for distant recurrence in HR+/HER2− and triple-negative BC patients was 80% (8/10) and 75% (3/4), respectively. In the two patients with ctDNA not detected at the time of distant recurrence, the sites of recurrence were bone only and lung with local axilla (Supplementary Table S2, https://doi.org/10.1016/j.esmoop.2025.104296).

**Table 1:** Overview of ctDNA detection by timepoint and clinical recurrence status

Time since diagnosis (no. of samples)	12 months (*n* = 9)	36 months (*n* = 15)	At recurrence (*n* = 19)	Patients with samples within 2 years from recurrence
Clinical status	Recurred later^a^	2 recurred later^a^ 13 no recurrence	Recurred	Recurred
Sensitivity (distant)	3/4 (75%)	1/2 (50%)	10/12 (83%)^b^	11/13 (85%)
Sensitivity (local)	0/5 (0%)		1/7 (14%)	1/8 (13%)
Specificity		13/13 (100%)		

ctDNA, circulating tumor DNA.

a Samples were collected at a median of 9.6 months before clinical detection of recurrence (range 1.4-32.1 months). Four patients with a sample available before clinical detection of distant recurrence had ctDNA detected with lead times of 3.4, 9.0, 10.1, and 18.5 months.

b One ctDNA+ patient and one ctDNA− patient did not have a second sample collected at the time of recurrence; hence 14 total patients experienced distant recurrence and 11 (79%) had ctDNA detected at or before the time of clinical recurrence detection.

We analyzed plasma samples from eight patients with local recurrence or new primary BC. One patient had ctDNA detected with contralateral primary BC, leading to a sensitivity for detection of localized BC of 13% (Table 1). For one patient with local recurrence, a sample collected before detection of local recurrence yielded a ctDNA-negative result.

In patients with no evidence of disease recurrence, all 13 samples at 36 months post-diagnosis were ctDNA negative [specificity 100% (13/13)]. The positive predictive value (PPV) for any recurrence was 100% (12/12); all 15 ctDNA-positive samples were from 11 patients with distant recurrence and 1 patient with local recurrence (Table 1).

### Genomic and epigenomic analysis

Of the 15 ctDNA-positive samples, 9 were based on methylation calls only, 1 on genomic calls only, and 5 had both. Therefore, the addition of methylation calls increased detection by a factor of 2.5, from 6 to 15 positive samples.

There were eight alterations consistent with BC carcinogenesis and acquired treatment resistance that were identified in six samples with genomic calls (Supplementary Table S3, https://doi.org/10.1016/j.esmoop.2025.104296). An *ESR1* E380Q mutation was identified in a patient with endocrine therapy resistance at the time of recurrence, confirming the ability of the assay to identify acquired resistance mutations in the adjuvant treatment setting.

### ctDNA quantitation

**Figure 3:**
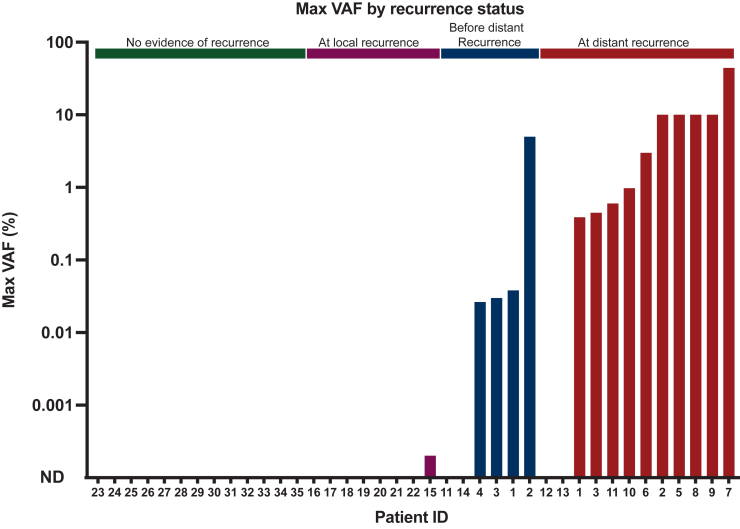
**VAF by recurrence status.** The lowest estimated ctDNA level was detected in a patient with local recurrence (0.0002%). The median estimated ctDNA level was 0.034% (range 0.026%-5.0%) in 5 samples collected before distant recurrence and 6.5% (range 0.39%-44.3%) in 12 samples collected at the time of distant recurrence. ctDNA, circulating tumor DNA; ND, not detected; VAF, variant allele fraction.

ctDNA quantity rose with increasing burden of cancer (Figure 3). The maximum VAF per sample was used to calculate the median value for each of the five groups: ctDNA was not detected in patients without recurrence or in seven of the eight patients who later had local recurrence. The lowest ctDNA level was detected in a sample at the time of local recurrence (0.0002% VAF). Before distant recurrence the median maximum VAF was 0.034% (range 0.026%-5.0%), while at the time of distant recurrence the median maximum VAF was 6.5% (range 0.39%-44.3%).

## Discussion

This is the first study to demonstrate the feasibility of MRD detection in EBC using a plasma-only multiomic ctDNA-based approach. The Guardant Reveal assay demonstrates high sensitivity and specificity to detect distant BC recurrence, including identification of ctDNA-positive samples up to 18.5 months ahead of clinical relapse and identification of a therapy-related resistant mutation at the time of clinical progression (*ESR1* E380Q).

Most ctDNA assays for MRD detection in EBC require prior knowledge of the primary tumor’s genomic profile, requiring tissue for whole-exome sequencing.^9^, ^10^, ^11^, ^12^, ^13^, ^14^, ^15^, ^16^ This study’s plasma-only assay incorporates epigenomic analysis, achieving a 2.5 times greater detection rate than genomic analysis alone, obviating the need for tissue sampling. Sensitivity of ctDNA assays for detecting recurrence in BC varies widely (19%-100%)^9^^,^^10^^,^^14^^,^^19^ and depends on timing and frequency of sample collection relative to clinical detection of recurrence. Serial sampling closer to clinical recurrence generally achieves higher sensitivity (≥70%), but rates are lower for local recurrence and distant metastases limited to central nervous system, lung, or bone compared with other sites. Sensitivity may also differ by cancer subtype, as more indolent tumors shed less ctDNA. Specificity (94%-100%) and PPV (76%-100%) are generally high, but may decrease if samples are taken before systemic therapy completion, as therapy may clear ctDNA/MRD in some patients. Optimal timing and frequency of MRD testing in BC is not yet established.

Removing the need for tissue in MRD testing provides logistical advantages, since studies show that up to 41% of EBC patients lack sufficient archived tumor material for tumor-informed MRD assays,^9^^,^^11^^,^^12^^,^^14^ particularly after neoadjuvant therapy. Thus, a plasma-only ctDNA approach improves feasibility in settings where tumor tissue is not readily available.

Detecting MRD or molecular progression before clinical recurrence offers an opportunity to modify patient management for meaningful clinical benefits. The PADA-1 trial demonstrated the potential of such an approach in MBC: in patients with rising *ESR1* mutations in cfDNA and absence of radiographic evidence of disease progression, switching from an aromatase inhibitor to fulvestrant while continuing palbociclib resulted in improved progression-free survival.^20^ This highlights the clinical utility of ctDNA-monitoring for early endocrine therapy resistance. Similar strategies need to be explored in EBC to demonstrate the utility of MRD monitoring to direct earlier imaging and/or targeted systemic therapy in adjuvant and surveillance settings.

This study demonstrated the feasibility of MRD detection in BC using a plasma-only multiomic ctDNA-based approach, demonstrating high specificity and sensitivity for distant recurrence required for clinical application. However, the small sample size in this pilot study was a key limitation, requiring larger studies in EBC populations to validate the assay’s performance and its applications in EBC management. Another limitation was the lack of paired samples in some patients, as seven had ctDNA detected at recurrence but lacked prior samples to assess pre-recurrence detection of ctDNA. Additionally, the lead time may be underestimated, as samples before 12 months post-diagnosis were not tested.

In summary, our findings highlight the potential of a plasma-only ctDNA assay in detecting MRD and identifying BC recurrence risk ahead of clinical relapse, with high specificity and sensitivity enabled by integrating epigenomic and genomic analyses. These results set the stage for earlier targeted and thus more effective surveillance and treatment strategies.

## References

[1] Jemal A., Ward E.M., Johnson C.J. (2017). Annual report to the nation on the status of cancer, 1975-2014, featuring survival. J Natl Cancer Inst.

[2] Ferlay J., Colombet M., Soerjomataram I. (2019). Estimating the global cancer incidence and mortality in 2018: GLOBOCAN sources and methods. Int J Cancer.

[3] Pan H., Gray R., Braybrooke J. (2017). 20-Year risks of breast-cancer recurrence after stopping endocrine therapy at 5 years. N Engl J Med.

[4] Early Breast Cancer Trialists’ Collaborative Group (EBCTCG) (2005). Effects of chemotherapy and hormonal therapy for early breast cancer on recurrence and 15-year survival: an overview of the randomised trials. Lancet Lond Engl.

[5] Cardoso F., Kyriakides S., Ohno S. (2019). Early breast cancer: ESMO Clinical Practice Guidelines for diagnosis, treatment and follow-up. Ann Oncol.

[6] National Comprehensive Cancer Network NCCN Guidelines: Breast Cancer (Version 3.2022). https://www.nccn.org/professionals/physician_gls/pdf/breast.pdf.

[7] Oltra A., Santaballa A., Munárriz B., Pastor M., Montalar J. (2007). Cost-benefit analysis of a follow-up program in patients with breast cancer: a randomized prospective study. Breast J.

[8] Kokko R., Hakama M., Holli K. (2005). Follow-up cost of breast cancer patients with localized disease after primary treatment: a randomized trial. Breast Cancer Res Treat.

[9] Magbanua M.J.M., Swigart L.B., Wu H.-T. (2021). Circulating tumor DNA in neoadjuvant-treated breast cancer reflects response and survival. Ann Oncol.

[10] Coombes R.C., Page K., Salari R. (2019). Personalized detection of circulating tumor DNA antedates breast cancer metastatic recurrence. Clin Cancer Res.

[11] Parsons H.A., Rhoades J., Reed S.C. (2020). Sensitive detection of minimal residual disease in patients treated for early-stage breast cancer. Clin Cancer Res.

[12] Lipsyc-Sharf M., de Bruin E.C., Santos K. (2022). Circulating tumor DNA and late recurrence in high-risk hormone receptor-positive, human epidermal growth factor receptor 2-negative breast cancer. J Clin Oncol.

[13] Garcia-Murillas I., Schiavon G., Weigelt B. (2015). Mutation tracking in circulating tumor DNA predicts relapse in early breast cancer. Sci Transl Med.

[14] Garcia-Murillas I., Chopra N., Comino-Méndez I. (2019). Assessment of molecular relapse detection in early-stage breast cancer. JAMA Oncol.

[15] Li S., Lai H., Liu J. (2020). Circulating tumor DNA predicts the response and prognosis in patients with early breast cancer receiving neoadjuvant chemotherapy. JCO Precis Oncol.

[16] Lin P.-H., Wang M.-Y., Lo C. (2021). Circulating tumor DNA as a predictive marker of recurrence for patients with stage II-III breast cancer treated with neoadjuvant therapy. Front Oncol.

[17] Silva J.M., Silva J., Sanchez A. (2002). Tumor DNA in plasma at diagnosis of breast cancer patients is a valuable predictor of disease-free survival. Clin Cancer Res.

[18] De Gregorio A., Nagel G., Möller P. (2020). Feasibility of a large multi-center translational research project for newly diagnosed breast and ovarian cancer patients with affiliated biobank: the BRandO biology and outcome (BiO)-project. Arch Gynecol Obstet.

[19] Janni W., Huober J., Huesmann S. (2021). 144P A personalised sequencing approach for liquid biopsy-based detection of recurrent disease in early-stage breast cancer. Ann Oncol.

[20] Bidard F.-C., Hardy-Bessard A.-C., Dalenc F. (2022). Switch to fulvestrant and palbociclib versus no switch in advanced breast cancer with rising ESR1 mutation during aromatase inhibitor and palbociclib therapy (PADA-1): a randomised, open-label, multicentre, phase 3 trial. Lancet Oncol.

