# Supplementary material for: Detection of minimal residual disease and prediction of recurrence in breast cancer using a plasma-only circulating tumor DNA assay
Source: ESMO Open. 2025 Mar 22;10(4):104296. doi: 10.1016/j.esmoop.2025.104296 (PMC11982450; doi:10.1016/j.esmoop.2025.104296)
Supplement: Supplementary data [file mmc1.docx]

**Supplementary Material**

**Table 1. Clinical Study Cohort Characteristics**

|  | Total n (%) | No. with Distant Recurrence | No. with Local Recurrence |
| --- | --- | --- | --- |
| **Histological Subtype** |  |  |  |
| HR+/HER2- | 25 (71%) | 10 | 6 |
| HR+/HER2+ | 2 (6%) | 0 | 1 |
| TNBC | 8 (23%) | 4 | 1 |
| **Pathologic Stage^1^** |  |  |  |
| I | 9 (26%) | 4 | 0 |
| II | 13 (37%) | 3 | 6 |
| III | 3 (9%) | 3 | 0 |
| Non-pCR post NACT | 8 (23%) | 4 | 2 |
| pCR post NACT | 2 (6%) | 0 | 0 |
| **Grade** |  |  |  |
| 2 | 22 (63%) | 9 | 6 |
| 3 | 13 (37%) | 5 | 2 |
| **Total Patients** | **35** | **14** | **8** |
| 1 – AJCC VII edition  **Abbreviations:** NACT – neoadjuvant chemotherapy; pCR – pathologic complete response | | | |

**Table 2. Clinical Characteristics of Patients with Distant Recurrence and Association with ctDNA Detection at or Before the Time of Clinical Detection of Recurrence**

| PATIENT | RECEPTOR SUBTYPE | TREATMENT | TIME FROM DIAGNOSIS TO RECURRENCE (MONTHS) | SITE(S) OF RECURRENCE | CTDNA DETECTED? | TIME FROM BLOOD COLLECTION TO RECURRENCE (MONTHS) |
| --- | --- | --- | --- | --- | --- | --- |
| 1 | HR+/HER2- | Chemotherapy | 15.6 | Liver | Yes | 3.4, -0.4 |
| 2 | HR+/HER2- | Endocrine therapy | 20.7 | Axilla, Lung, Ovary | Yes | 9.0, -0.4 |
| 3 | HR+/HER2- | Endocrine therapy | 22.7 | Bone, Liver | Yes | 10.1, -1.8 |
| 4 | HR+/HER2- | Endocrine therapy | 54.4 | Bone, Lung, Axilla | Yes | 18.5 |
| 5 | TNBC | Chemotherapy | 6.7 | Liver | Yes | -0.3 |
| 6 | TNBC | Chemotherapy | 9.3 | Liver, Lung | Yes | -0.4 |
| 7 | TNBC | Chemotherapy | 13.2 | Liver | Yes | -0.5 |
| 8 | HR+/HER2- | Endocrine therapy | 19.4 | Bone, Liver, Lung | Yes | -1.3 |
| 9 | HR+/HER2- | Chemotherapy + Endocrine therapy | 23.3 | Bone, Liver | Yes | -0.4 |
| 10 | HR+/HER2- | Endocrine therapy | 21.5 | Bone | Yes | -4.3 |
| 11 | HR+/HER2- | Endocrine therapy | 39.0 | Bone, Liver | Yes | 26.1 (ND), -0.4 |
| 12 | HR+/HER2- | Chemotherapy | 10.6 | Bone | No | 0.0 |
| 13 | TNBC | Chemotherapy | 3.8 | Axilla, Lung | No | -0.7 |
| 14 | HR+/HER2- | Endocrine therapy | 69.9 | Lymph nodes | No | 32.1 |

____________________________________________________________________________________________________________

**Table 3. Identified genomic alterations were consistent with breast cancer carcinogenesis and acquired treatment resistance**

| **Alteration** | **Number of Observations** | **VAF** |
| --- | --- | --- |
| Patient 1 (2 samples) |  |  |
| *ERBB2* (G778_P780dup) | 2 | 0.04% - 1.1% |
| *MAP3K1* (L920 frameshift) | 1 | 0.39% |
| Patient 3 |  |  |
| *GATA3* (splice variant Indel) | 1 | 0.45% |
| Patient 7 |  |  |
| *AKT1* (splice variant E17K) | 1 | 48.50% |
| *RB1* (R661W) | 1 | 44.30% |
| Patient 9 |  |  |
| *ESR1* (E380Q) | 1 | 0.31% |
| Patient 10 |  |  |
| *KRAS* (G12V) | 1 | 1.70% |
| *PIK3CA* (H1047R) | 1 | 0.98% |

VAF: variant allele fraction
